# Next Chapter in the Legend of Silphion: Preliminary Morphological, Chemical, Biological and Pharmacological Evaluations, Initial Conservation Studies, and Reassessment of the Regional Extinction Event

**DOI:** 10.3390/plants10010102

**Published:** 2021-01-06

**Authors:** Mahmut Miski

**Affiliations:** Department of Pharmacognosy, Faculty of Pharmacy, Istanbul University, Istanbul 34116, Turkey; mahmud.miski@istanbul.edu.tr or mahmut.miski@gmail.com; Tel.: +90-545-550-4455

**Keywords:** silphion, *Ferula*, *F. drudeana*, medicinal oleo-gum-resin, chemistry and pharmacology, extinction, conservation

## Abstract

Silphion was an ancient medicinal gum-resin; most likely obtained from a *Ferula* species growing in the Cyrene region of Libya ca. 2500 years ago. Due to its therapeutic properties and culinary value, silphion became the main economic commodity of the Cyrene region. It is generally believed that the source of silphion became extinct in the first century AD. However, there are a few references in the literature about the cultivated silphion plant and its existence up to the fifth century. Recently, a rare and endemic *Ferula* species that produces a pleasant-smelling gum-resin was found in three locations near formerly Greek villages in Anatolia. Morphologic features of this species closely resemble silphion, as it appears in the numismatic figures of antique Cyrenaic coins, and conform to descriptions by ancient authors. Initial chemical and pharmacological investigations of this species have confirmed the medicinal and spice-like quality of its gum-resin supporting a connection with the long-lost silphion. A preliminary conservation study has been initiated at the growth site of this rare endemic *Ferula* species. The results of this study and their implications on the regional extinction event, and future development of this species will be discussed.

## 1. Introduction

Silphion (=*Silphium*) refers to an oleo-gum-resin of a well-known ancient medicinal plant [1]. Due to its numerous therapeutic uses [2,3] and spice qualities [4], it was the major economic resource of the Cyrenaic region of Libya [5] for six centuries. The kings of Cyrene endeavored to retain the monopoly of silphion trade, nevertheless, a certain amount was smuggled to the Carthaginians, and Carthage became a supplier of silphion to some extent [6]. Although wide-spread trading of silphion in the Mediterranean region was initiated with the establishment of the Greek colonization of the Cyrenaic region, the commercial source must have been well developed prior to these large-scale trading practices and there is even some fragmented evidence in Minoan pictograms that suggests the presence of silphion production in the Late Bronze Age [1,7,8]. Since the natural habitat of the silphion plant was near Egypt, one might wonder about the awareness of ancient Egyptians regarding the presence and medicinal values of the silphion plant. Indeed, there is some archaeological and linguistic evidence indicating that ancient Egyptians knew about silphion and used it in Egyptian rituals such as the renewal of the King’s vigor in the Old Kingdom [1,8].

Dedicated to the memory of victims of COVID-19 pandemic.

Preliminary data was presented at the IXth Apiales Symposium on 1 August 2017, in Guangzhou, China.

Most ancient scientists have mentioned silphion in their manuscripts, including Theophrastus of Eresos, father of Botany, who provided some morphological description of the plant in his “Enquiry into Plants” [9]. Hippocrates extensively used silphion in his recipes [8], for example, he reported preparation of a poultice made with silphion to treat a protruded gut (i.e., hernia) in his book “On Fistulae” [10]. Soranus of Ephesus advised drinking of a chick-pea sized Cyrenaic balm (i.e., silphion) once a month with two cyaths of water to induce the menstruation in his “Gynecology” [11]. Asclepiades of Bithynia described an oral prescription as well as a plaster containing silphion for the treatment of cardiac disease defined as “inflammation in the heart” [12]. Pliny the Elder described an early account of the extinction of the silphion plant in his “Natural History” as well as thirty-nine remedies made with silphion [3,13]. Dioscorides dedicated a comprehensive monograph in his “De Materia Medica” for this drug. In addition to the description of the silphion plant [14], he provided a detailed documentation of its medicinal uses for the treatment of several diseases such as goiter, sciatica, tooth ache, intestinal disorders, hormonal disorders, epilepsy, tetanus, polyps, and malignant tumors in his monograph [15]. Ironically, the consensus of the experts in classical studies is that the only direct source of information about the silphion plant is Theophrastus, and that later descriptions were commentary and none of the other ancient scientists had actually seen the plant itself [16]. According to some medicinal historians, silphion was used as an aphrodisiac [2] and as a powerful contraceptive [17]. Perhaps due to these virtues, silphion was over harvested and became extinct in the first century AD [17,18]. However, there are some references suggesting that silphion was cultivated and continued to exist well into the fifth century. Synesius, a Cyrenian aristocrat and bishop of Ptolemais, claimed that he had seen the plant itself and sent lots of silphion juice to his friend Tryphon in Constantinople (i.e., now Istanbul, Turkey) [19]. Despite the extinct status of silphion, it is still a hot research topic among the academicians, and several master and doctorate theses have been dedicated to this subject [20,21,22].

Unfortunately, no specimen of the silphion plant exists to confirm its genus or family, but descriptions provided by Theophrastus in the “Enquiry into Plants” [9] and descriptions of the other ancient scientists strongly suggest that this plant, most likely, is a member of the genus *Ferula* [16,23]. *Ferula* (Apiaceae) comprises ca. 190 species worldwide and includes well known medicinal plants such as *F. assa-foetida* L. and *F. gummosa* Boiss. that are sources of the medicinal oleo-gum-resins asafoetida and galbanum, which have been used since ancient times. In addition to silphion, Dioscorides also described other oleo-gum-resins obtained from *Ferula* species such as ammoniakon (African ammoniacum), sagapenon (*sagapenum*), narthex and chalbane (*galbanum*) in his De Materia Medica [13].

**Figure 1:**
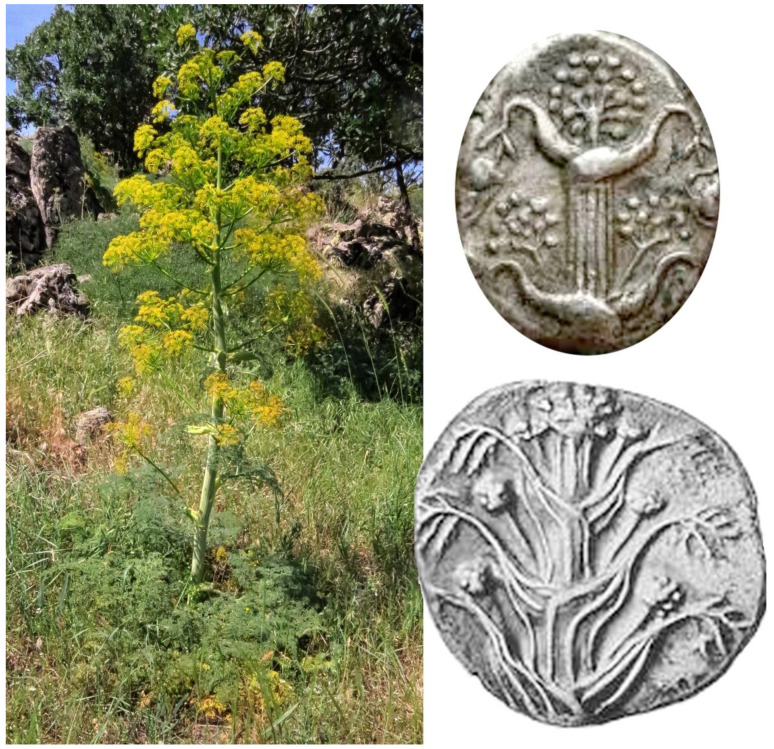
Comparison of the general appearance of the Turkish endemic *Ferula* species with those of numismatic figures on Cyrenaic coins.

A rare and endemic species of *Ferula* growing near Central Anatolia closely resembles the description and numismatic figures of silphion (Figure 1). Furthermore, the organoleptic qualities of its oleo-gum-resin exudate are also in close agreement with the description of silphion juice by Dioscorides [13]. Preliminary phytochemical analyses of the resin indicate the presence of highly complex sesquiterpenoids and coumarins with novel structures as well as other, known compounds. The known compounds have been previously described from other medicinal plants such as sweet flag (i.e., *Acorus calamus* L.), *galbanum* (i.e., *Ferula gummosa* Boiss.), rosemary (i.e., *Rosmarinus officinalis* L.), sage (i.e., *Salvia* sp.) and artichoke (i.e., *Cynara scolymus* L.). The known biological activities of these compounds corroborate the medical uses of silphion mentioned in the monograph of Dioscorides and other sources [2,13,14].

Only small populations of the endemic *Ferula* species, here considered to represent the silphion plant, exist in three locations in Anatolia all associated with the locations of former Greek villages. These locations were discovered serendipitously with the help of local villagers, and these plant populations grow in protected enclaves such as stone-walled orchards. No other populations have been found to exist except for a small population found in an adjacent lot of the original plant material collection site. Conservation studies have been initiated to preserve and propagate this species. Details of the initial conservation study and training of the local villagers to protect this plant species while providing them potentially valuable economic resource will be discussed. 

## 2. Materials and Methods

### 2.1. Plant Material

The *Ferula* species was first collected by Walter E. Siehe, a German engineer and plant collector [24], in July 1909 in the northern Adana province of South Anatolia. Siehe identified this species as *Ferula ovina* Boiss. and sent plant specimens to the Herbarium of the Komarov Botanical Institute in Leningrad (LE), USSR, and Herbarium of Royal Botanical Garden, Edinburgh (RBGE), UK. In 1930, during the examination of herbarium specimens at LE, Korovin identified this plant as a new species and named it as *Ferula drudeana*. The first description of *F. drudeana* was published by Korovin without any field observation of this plant in his “Generis Ferula (Tourn.) L. Monographia Illustrata” in 1947 [25]. In 1983, we found *F. drudeana* Korovin near Hasan Dağı in the Cappadocia region of Anatolia while collecting *Ferula* specimens for investigation of their biologically active secondary metabolites. The plant voucher was deposited in the Herbarium of the Faculty of Pharmacy of Istanbul University (ISTE 50880) and identified by Dr. E. Tuzlacı. In 2004, *F. drudeana* was also rediscovered where it was originally collected by Siehe in 1909 [26]. Although the botanical descriptions of *F. drudeana* have been published by both Korovin and, M. Sağıroğlu and H. Duman [25,26], these descriptions omit macro-morphologic details that provide important clues about the relation of this species to the silphion plant. The roots of wild growing *F. drudeana* were recollected from the Cappadocia region near Hasan Dağı in May 2019 for reinvestigation of its secondary metabolites and herbarium specimen was deposited in the Herbarium of the Faculty of Pharmacy of Istanbul University (ISTE 116411).

### 2.2. General Experimental Procedures, Extraction and Isolation of Secondary Metabolies

Optical rotations were measured using a Perkin-Elmer Model 241 polarimeter in a 100 × 2 mm cell in CHCl_3_. IR spectra (neat) were recorded on a Beckman IR 4230 spectrophotometer. NMR spectra were acquired on a Bruker WM-400 spectrometer operating at 400 MHz for ^1^H- and 100.6 MHz for ^13^C-NMR spectra, and were referenced to the residual deuterated solvent peaks. EI-MS spectra were recorded on a Varian MAT 711 spectrometer (70 eV, direct inlet), CI-MS were recorded on a Varian MAT 44 spectrometer (isobutane used as ionization gas).

Air-dried and coarsely powdered roots (900 g) of *Ferula drudeana* were extracted successively in a Soxhlet extractor with hexane, dichloromethane and methanol in the following manner; plant material was briefly soaked (ca. 10 min) in the solvent at room temperature and then the solvent was allowed to siphon. The room temperature maceration procedure was repeated three times, and the solvent of combined plant extracts was distilled on a rotary evaporator in vacuo. Following the room temperature maceration procedure, additional solvent was charged on the plant material and continuous extraction procedure was allowed to continue for 24 h in the Soxhlet extractor. The plant material was removed and dried completely after each extraction step before continuing the extraction with more polar solvents (i.e., dichloromethane and methanol). After the completion of the sequential extraction process, extracts of each solvent were combined and concentrated using a rotary evaporator in vacuo. The yields of each extract as follows; hexane extract 46 g (5.1%), dichloromethane extract 18 g (2.0%) and methanol extract 68.5 g (7.6%).

Hexane and dichloromethane extracts (10 g each) were fractionated over a silica gel column (5 × 60 cm) using hexane-ethyl acetate mixtures as elution solvent. If necessary, further separation and purification of compounds were carried out on a Sephadex LH-20 column (4 × 65 cm) packed in hexane/dichloromethane/methanol (14:9:1) followed by prep. TLC (1 mm thickness, silica gel F254 developed with hexane/ethyl acetate at 9:1, 7:3, or 1:1 mixtures).

## 3. Results and Discussions

### 3.1. Comparison of Morphological/Organoleptic Characteristics

Since the majority of the known morphological descriptions of silphion were reported by Theophrastus [9] and Pliny the Elder [13], their descriptions will be used to illustrate the similarities between the parts of silphion plant and those of *Ferula drudeana*. In addition, despite their exaggerated representations, numismatic figures of the silphion plant on the Cyrenaic coins were utilized to compare the macro-morphology of *F. drudeana* to the silphion plant. 

#### 3.1.1. General Appearance

**Figure 2:**
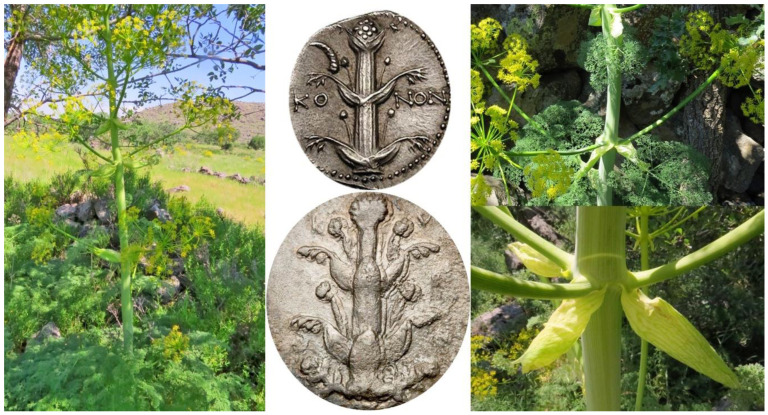
Opposite arrangement of the inflorescence branches and leaves/sheaths of *Ferula drudeana*.

**Figure 3:**
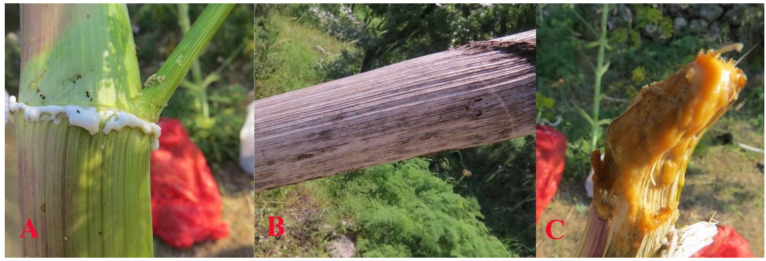
(**A**) Oleo-gum-resin exudate oozing from the injured stem of *Ferula drudeana*. (**B**) Resin channels easily observable on the dried stalk of plant. (**C**) Dried resin accumulated at the tip of a broken stem.

Based on the frequently depicted numismatic figures, one of the most distinct morphological characters of silphion is the opposite arrangement of stem leaves, which is very rarely observed with the other *Ferula* species. Undoubtedly, a predominant feature of *F. drudeana* observed in the field was the opposite arrangement of inflorescence branches (Figure 2), as depicted for the silphion plant on Cyrenaic coins. Occasionally, three or more sheaths of stem leaves appeared at the same stem node, especially at the base of flowering branches in the upper section of the plant. The stalk of silphion was often represented with a ribbed appearance on the numismatic figures. Although the stem of *Ferula drudeana* does not have such protruded ribs, it has a striated appearance due to the presence of resin channels under the surface epidermis of the stem (Figure 3).

#### 3.1.2. Fruits (Phyllon)

**Figure 4:**
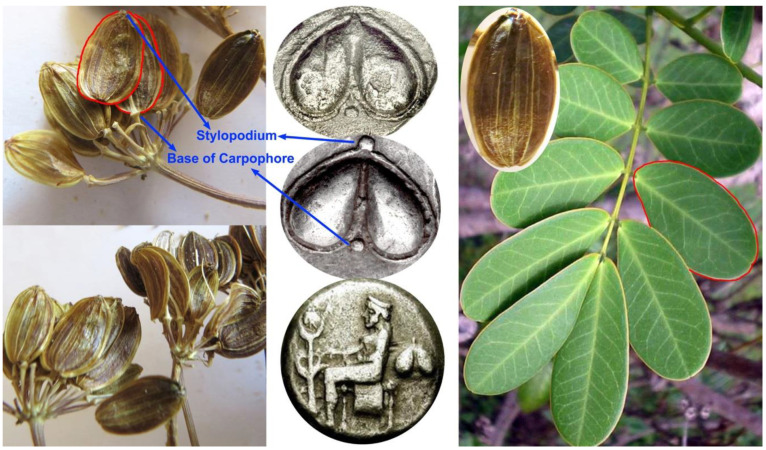
Comparison of the fruits of *F. drudeana* with the numismatic figures, shape of the mericarps closely resembles leaflets of pinnatipartite leaves.

Theophrastus calls the fruits of silphion leaf-like (i.e., phyllon); indeed, mericarps of the *Ferula drudeana* fruits remarkably resemble the leaflets of a pinnatipartite leaf (Figure 4). In most of the literature, the silphion fruit is described and positioned as a “heart-shaped” fruit [23]. However, careful examination of the numismatic figures of fruits reveals that the “heart-shape” was actually formed by the overlapping mericarps of silphion fruits with a clear depiction of the stylopodium at the top and base of carpophore at the bottom appearing between them. As with the case of extant *Ferula* fruits, *F. drudena*’s fruit is a schizocarpic fruit but each mature mericarp has a papery resemblance (Figure 4) which would facilitate the spreading of mericarps by wind, as described by Theophrastus; “when a strong south wind blows after setting of the dog-star, it is scattered abroad and the silphium grows from it” [9].

#### 3.1.3. Oleo-Gum-Resin (Silphion)

Dioscorides described the oleo-gum-resin of the silphion plant as the juice of the plant [14,15]. According to Theophrastus and Pliny the Elder [3,9], there were two kinds of juice obtained from the silphion; stalk juice and root juice which were produced by the incision of stem or root of plant (Figure 3), respectively. Dioscorides described the juice of silphion as the most effective part of silphion, indeed, the oleo-gum-resin of the plant contains virtually all of the bioactive chemicals produced by the plant, which justifies the statement of Dioscorides. Dioscorides also reiterated that the smell of resin is predominant and gentle. In contrast, the resins obtained from Syrian and Median (i.e., Persian) plants were weaker in activity and had a poisonous smell. Such description sets silphion clearly apart from the resins produced by the other *Ferula* species such as *F. assa-foetida* These descriptions closely match the organoleptic characteristics of the oleo-gum-resin of *F. drdeana*. It has a very pleasant-smelling odor but an acrid taste due to the high terpenoid content of the resin.

#### 3.1.4. Roots

**Figure 5:**
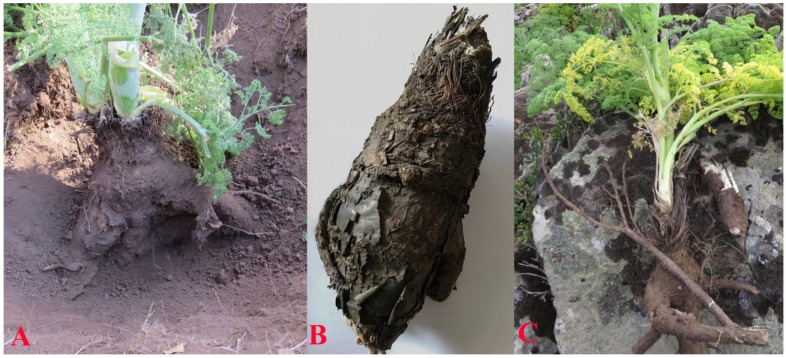
(**A**) Partially excavated root of *Ferula drudeana*. (**B**) Soil cover removed root reveals the dark-brown/black color of bark. (**C**) Fully excavated root length exceeds 60 cm.

According to Theophrastus of Eresos, silphion had a thick root with black bark and grows to the length of a cubit (ca. 46 cm) or a little longer [9]. The root of *F. drudeana* is very wide, often as wide as. 20–25 cm at the top section (Figure 5). As described by Theophrastus, the color of the root bark was black and the length of the fully excavated root of silphion is well over a cubit in length. In addition, Pliny the Elder in his Natural History [13], described the roots of silphion as numerous and thick. Consistent with this description the root of *F. drudeana* always divides into several thick branches ca. 20 cm below the basal leaves of the plant under the ground.

#### 3.1.5. Leaves (Maspetum)

Pliny the Elder described the leaves of silphion as: “The leaves of this plant were known as “maspetum,” and bore considerable resemblance to parsley” in his “Natural History” [13]. The leaf segments of *Ferula drudeana* are not as broad as the typical parsley leaf segment. This difference may be due to the ecological transformation of *F. drudeana* in its current location over the 2000 years of growth cycles, and perhaps, *F. drudeana* is an ecotype of the silphion plant. An alternate explanation of this discrepancy could be due to the morphological variations of basal leaf segments of *Ferula* species; there are several examples of such variations known from other *Ferula* species, such as *F. tingitana* L. and *F. elaeochytris* Korovin [27,28].

**Figure 6:**
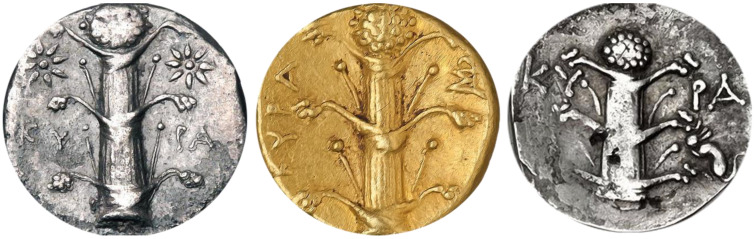
Examples of Cyrenaic coins with the phallic representation of silphion plant.

Because of the vast variations of numismatic figures of silphion plant, comparison of the morphological features of *Ferula drudeana* beyond the aforementioned characters may not be accurate. Some authors believe that some of the silphion numismatic figures do not reflect the real representation of source plant but simply serve as an exaggerated advertisement [29]. For example, a typical advertising numismatic figure of silphion plant often appears as a phallic representation on Cyrenaic coins (Figure 6).

### 3.2. Phylogenetic Classification Analyses of Ferula drudeana

Extensive phylogenetic analyses of *Ferula dureana* were carried out [30] to elucidate its taxonomic position. Based on the results of nrDNA ITS tree analysis *F. drudeana* was classified in the same group as *F. huber-morathii* Peşmen another endemic species of Anatolia. On the other hand, concatenated pDNA tree analysis places *F. drudeana* in a completely different position that has no direct relation to *F. huber-morathii* but is closer to two other Anatolian endemic *Ferula* species (i.e., *F. anatolica* Boiss. and *F. coskunii* Sağıroğlu and Duman). However, the best maximum likelihood tree inferred from the 148 ITS sequences placed *F. drudeana* in the same group with *F. szowitsiana* DC. Despite the application of sophisticated molecular-level analyses, none of those taxonomic classifications appear to be consistent with the traditional systematic taxonomy and chemotaxonomic classification of *F. drudeana* [25].

### 3.3. Preliminary Chemical, Biological and Pharmacological Evaluations

**Figure 7:**
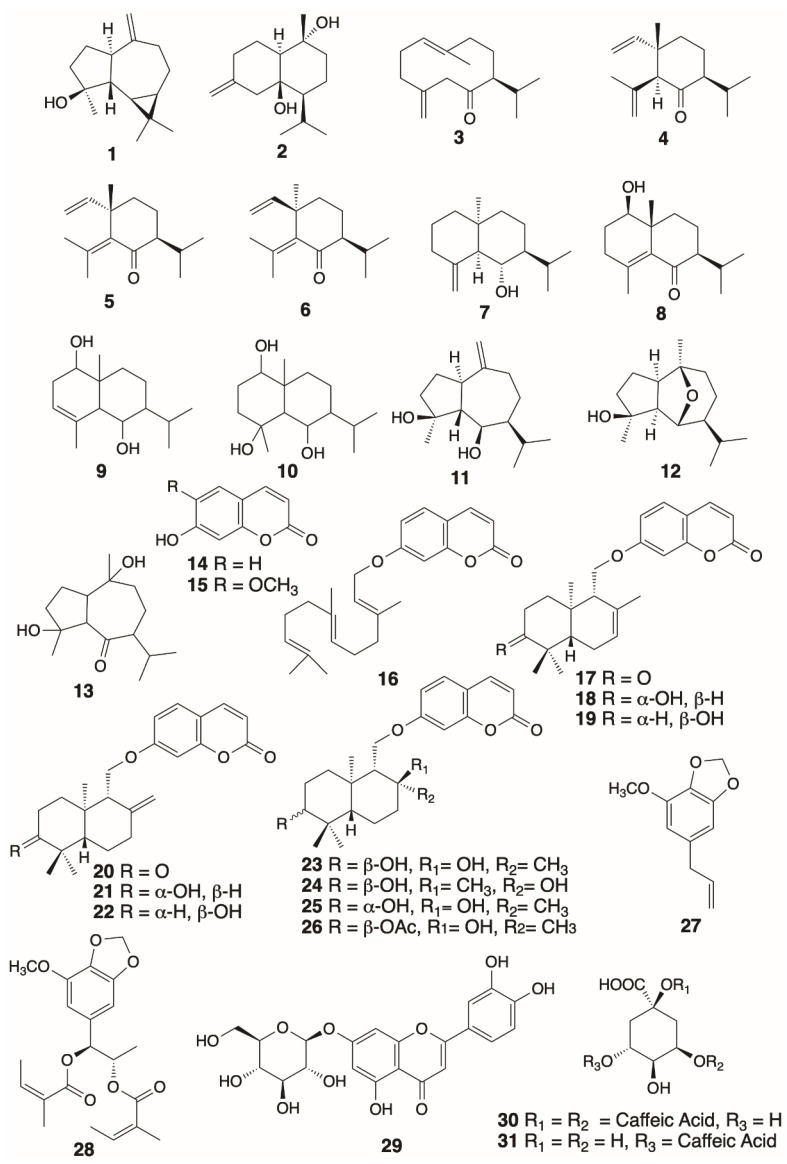
Chemical structures of the biologically active secondary metabolites isolated from the roots of *Ferula drudeana* Korovin.

Investigation of the secondary metabolites of the roots of *F. drudeana* yielded about 30 compounds. Most of them are sesquiterpenoid compounds with various skeletal types, as well as coumarins, sesquiterpene coumarins and phenolic compounds (Figure 7). The secondary metabolites isolated from the root extracts of *F. drudeana* were as follows: an alloaromadendren sesquiterpenoid; spathulenol (**1**) [31,32,33,34], a cadinene type sesquiterpenoid, calamendiol (**2**) [35], a germacrane type sesquiterpenoid; preisocalamendiol (**3**) [35,36], elemene type sesquiterpenoids; shyobunone (**4**) [35], isoshyobunone (**5**) [35], epi-isoshyobunone (**6**) [37], eudesmane type sesquiterpenoids; 10-epijunenol (**7**) [38,39], acorusnol (**8**) [40,41], a eudesmane-diol (**9**), a eudesmane-triol (**10**), guaiane type sesquiterpenoids; teucladiol (**11**) [42], chrysothol (**12**) [43,44], a guaiane-ketoalcohol (**13**), simple coumarins; umbelliferone (**14**), scopoletin (**15**), sesquiterpenoid coumarins; umbelliprenin (**16**) [45,46], conferone (**17**) [47], feselol (**18**) [47], conferol (**19**) [47], badrakemone (**20**) [48], colladonin (**21**) [48], badrakemin (**22**) [48], samarcandin (**23**) [49], feshurin (**24**) [49], isosamarcandin (**25)** [49], samarcandin acetate (**26**) [50], phenylpropanoid compounds; myristicin (**27**) [51,52], laserine (**28**) [53]. 

In addition, HPLC-MS analyses of the methanol extract of *Ferula drudeana* roots confirmed the presence of luteolin-7-β-d-glucoside (cynaroside) (**29**), cynarin (**30**) and chlorogenic acid (**31**) as bioactive polar metabolites [54]. Chemical constituents of the *F. drudeana* were highly diverse; such chemical and pharmacological diversity is unique among the investigated *Ferula* species.

Some of the compounds isolated from *Ferula drudeana* have previously been isolated from other medicinal plants such as *Acorus calamus* [35], *Salvia* sp. [55], *Teucrium* sp. [42], *Ferula gummosa* [56], and *Rosmarinus officinalis* [57]. In addition to those known compounds, several unknown compounds were isolated from the extracts of *F. drudeana*. It should be noted that the major sesquiterpenes of *F. drudeana* were also minor compounds of *F. gummosa* (syn. *F. galbaniflua* Boiss, and Buhse) which required the use of a large amount of galbanum resin to isolate those compounds [39]. The attractive pleasant smell of the oleo-gum-resin of *F. drudeana* is probably induced by the GABA_A_ receptor modulating activity of the volatile sesquiterpene compounds of its oleo-gum-resin [58]. This influential effect of the resin may account for the discovery and establishment of silphion as a state trade commodity of the ancient Greek colonies in the Cyrenaic region. A literature survey of the biological activity of other known compounds indicates that some of the common medicinal uses of silphion such as anti-inflammatory [33,59], antiproliferative, antimycobacterial [33,34], immunomodulator [32] selective estrogen receptor modulator [60] (i.e., aphrodisiac and emmenagogue), cardioprotective [61] activities, etc. could be accounted for by these compounds (Table 1). The presence of both terpenoid and phenolic compounds with anti-inflammatory and antioxidant compounds in silphion validates the oral prescription and plaster medications proposed by Asclepiades of Bithynia for the treatment of “inflammation in the heart” condition [12]. Recently, the value of *Acorus calamus* as a source of promising bioactive compounds in prevention and treatment of cardiovascular diseases has been questioned, the only negative aspect of *A. calamus* was the presence of highly mutagenic α- and β-asarones in its extracts [62]. Since the extracts of *F. drudeana* practically contain most of the bioactive sesquiterpenoids of *A. calamus* without α- and β-asarones, it may be an ideal substitute for *A. calamus* as a source of precious bioactive compounds/extracts to prevent and treat cardiovascular diseases, and other medical conditions where *A. calamus* was used as a traditional folk medicine. Furthermore, a recent publication on the potential aphrodisiac effects of *F. drudeana* extract and its sesquiterpene coumarins also confirms the notorious use of silphion [63]. 

Several Apiaceae plants have been proposed to be the Cyrenaic silphion, such as *Feula tingitana* L. [1,2,16], *F. communis* L. [18], *Thapsia garganica* L. [1,2,147] and *Cachrys ferulacea* (L.) Calestani [147,148] were just a few examples to name. *Ferula tingitana* and *F. communis* along with the other associated North African/Mediterranean basin *Ferula* species (i.e., *F. tunetana* Pomel ex Batt., *F. marmarica* Asch. and Taub. ex Asch. and Schweinf., *F. glauca* L. and *F. sinaica* Boiss.) were taxonomically placed in the section *Anatriches* Korovin of Subgenus *Euferula* (Boiss.) Korovin in the Korovin’s monograph [25]. Typical chemical compounds of these species are highly oxygenated daucane esters and sesquiterpene coumarins [103,149,150,151,152,153,154,155]. Although oleo-gum-resins of these species contain biologically active sesquiterpenoids, the noxious smell of their resins does not suggest any relation of these species to silphion. An endemic chemotype of the *Ferula communis* associated with symbiotic interaction with a fungus has also been proposed as a potential source of silphion, however, such ecological interaction of *F. communis* can generate a deadly variety of this plant with the production of toxic 4-hydroxycoumarin derivatives [156,157]. Due to the presence of highly poisonous compounds in its resin, *Thapsia garganica* could not have been the source of silphion [158]. Based on its heart-shaped fruits and presence in the Cyrenaic region of Libya, *Prangos ferulacea* (L.) *Lindl.* (Syn., *Cachrys ferulacea*) was also suggested as the source of silphion plant [147]. However, this plant is widely distributed in the Mediterranean region and is not an endemic species of the Cyrenaic region. Furhermore, the presence of 3,5-nonadiyne, an acetylenic compound with endogenous nitric oxide inhibitor activity, in its essential oil [148] does not provide sufficient evidence for the wide variety medicinal use as in the case of silphion.

### 3.4. Archaeobotanical Connections

In 1979, “The Manchester Museum Mummy Project” investigations identified the use of “*galbanum*” oleo-gum-resin to secure bandages during the wrapping of the mummy. The investigation team arrived at this conclusion by the chromatographic comparison of various organic extracts of mummy bandages with *galbanum* extracts and, more specifically, with umbelliferone [159]. Recently, a similar discovery was made during the investigation of embalming materials of two Egyptian child mummies [160]. It is highly problematic to rely on umbelliferone, a ubiquitous plant coumarin, as a marker substance of *galbanum*. Nevertheless, umbelliferone is also one of the major coumarin of *F. drudeana* oleo-gum-resin that may indicate the use of silphion during the mummification process. Especially, the known use of silphion in “Egyptian ritual of renewal of the King’s vigor” [8] makes perfect sense for the application of silphion during the mummification process to prepare the deceased person for the afterlife.

In 2015, an ancient Roman necropolis was discovered ca. 30 km NE of Rome. In order to understand the lifestyle adopted by the Roman Imperial community of *Ager Curensis*, a combined approach of morphological and gas chromatography-mass spectrometry (GC-MS) analyses was applied on the dental plaques of skeletal remains to identify their edible and/or medicinal plant species use [161]. One of the sesquiterpene derivative identified during the GC-MS analyses was shyobunol and authors suggest that this compound should be considered as a potential biomarker of a *Ferula* species. This assumption was derived from the presence of shyobunol in the essential oil of *F. vesceritensis* Coss. et Dur. [162], an endemic *Ferula* species from Algeria. However, elemene sesquiterpenoids are not common secondary metabolites of *Ferula* species. There are five published essential oil analyses of *F. vesceritensis* in the literature and just one of them reports elemene sesquiterpene (i.e., shyobunol) as its major component (i.e., 18.1%) [162,163,164,165,166]. Furthermore, comparisons of essential oil analyses of *Ferula* species from Turkey and Iran indicate that out of 35 *Ferula* species analyzed by GC-MS, only one species, *F. drudeana*, contains a high level of elemene-type sesquiterpenoids [54,167,168]. Essential oil of *F. drudeana* contains ca. 65% shyobunone isomeric mixtures and the essential oil composition of major *F. drudeana* populations were consistent [168]. Nevertheless, the elemene-type sesquiterpenoid identified by the GC-MS analyses of dental plaques was mentioned as shyobunol, not shyobunone. This discrepancy may be explained by the chemistry of the derivatization procedure applied to the samples before the GC-MS analyses; the calculus samples were treated with 3% hydrochloric acid overnight and then extracted with hexane. Following the extraction and concentration of analysis samples, they were treated with Burgess’s reagent to produce dehydrated derivatives of elemene-type sesquiterpenoids which were subjected to GC-MS analyses. This derivatization procedure may yield the same derivatives for shyobunone(s) and shyobunol(s). Additionally, it should be noted that elemene-type sesquiterpenoids can isomerize to germacrane derivatives via Cope Rearrangement at high temperatures during the GC analyses which could muddle the results of such analytical procedures. Nevertheless, we may speculate that the person who had this elemene-type sesquiterpenoid in his/her dental plaque might have indulged a meal with silphion sauce [169] or orally taken a silphion containing medication before their demise.

### 3.5. Initial Conservation Studies

Theophrastus of Eresos suggested that silphion is a wild plant and avoids cultivated land in the “Enquiry into Plants” [9], yet he indicated that “digging around the root improves the quality of silphion due to the change of soil”; however, such a statement directly contradicts his previous comments. Pliny the Elder also emphasized the resistance of silphion to the cultivation by declaring that any attempted cultivation “will leave the spot where it has been sown quite desolate and barren” in his Natural History [13]. Moreover, Hippocrates mentions that cultivation attempts in Ionia and the Peloponnesus failed [8]. Nevertheless, letters of Synesius that describe his observation of the cultivated silphion plants [19] contradict Theophrastus’, Pliny’s and Hippocrates’ statements about the resistance of silphion plant to cultivation.

**Figure 8:**
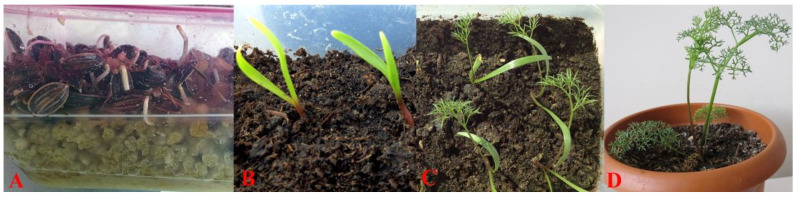
(**A**) Sprouting seeds of *Ferula drudeana*. (**B**) Seed leaves of *F. drudeana*. (**C**) First leaves of the seedlings of *F. drudeana*. (**D**) One-year old saplings of *F. drudeana*.

**Figure 9:**
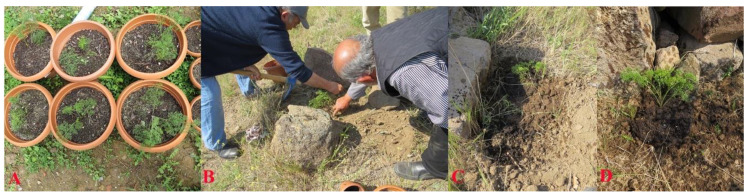
(**A**) Preparation of one-year and two-year old plants for transportation. (**B**) Planting in their parent plants’ location. (**C**) Replanted one-year old plant. (**D**) Replanted two-year old plant.

Since the local villagers’ attempt to grow *F. drudeana* from its seeds failed, seeds of *F. drudeana* were collected to initiate germination trials. Although a straight forward germination attempt of the seeds did not produce satisfactory results, application of contemporary seed germination procedures, such as cold stratification, successfully produced sprouting seeds (Figure 8). The seedlings of *F. drudeana* were generated from the sprouting seeds over the period of two subsequent years and then, both one- and two-year old plants were transported to the Cappadocia region for reintroduction and replanted in their parent plants’ location by local villagers (Figure 9) in May 2014. 

**Figure 10:**
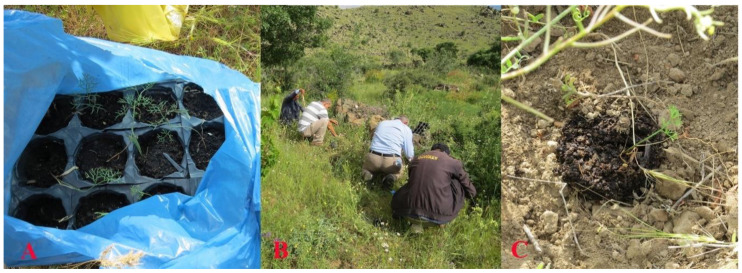
(**A**) Seedlings produced by the local villagers. (**B**) Planting of the seedlings. (**C**) A replanted seedling.

In addition to the transported plants, the villagers also planted the seedlings they had produced following the cold stratification germination procedure (Figure 10). Following the reintroduction of the saplings and seedlings of *F. drudeana*, their growth and development was followed by the local villagers. 

**Figure 11:**
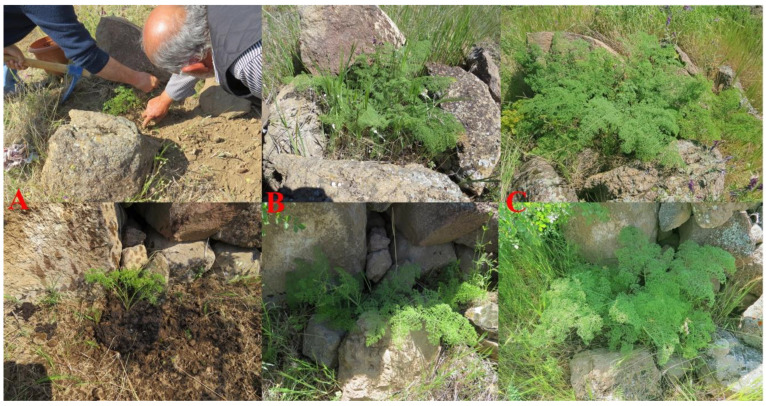
(**A**) Size of the basal leaves of saplings at the two-years old stage, (**B**) at the five-years old development stage of young plant, (**C**) at the seven-years old development stage of mature plant.

Despite the failure of establishment of seedlings and one-year-old saplings during the summer months, two-year old saplings were well established in the orchard and continued to grow in the following years. Over the next eight years, the basal leaves of re-established plants continued to grow to reach the maturation stage. However, they did not produce a fruiting stem. This unusually slow growth of the mature plant indicates that *Ferula drudeana* must be a monocarpic species, that is the development of a fruiting stem requires several years of rhizome development to reach such enormous size and then formation of the fruiting stem signals the end of *F. drudeana’s* life cycle. Comparison of the basal leaf size of the replanted plants at their eighth year of development stage with those of wild growing plants suggests that the fruiting stem of replanted *F. drudeana* might develop at the ninth or tenth year of the plant’s development stage (Figure 11).

**Figure 12:**
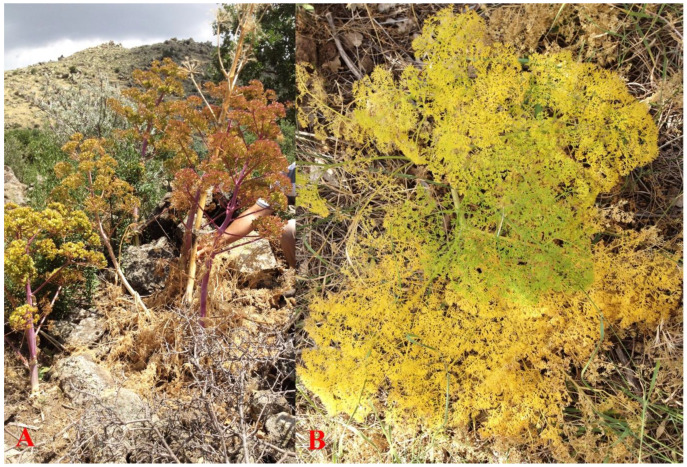
(**A**) Dying fruiting stem of *Ferula drudeana* and a dried stem. (**B**) A golden colored compound pinnate basal leaf of *F. drudeana*.

Following the production of a fruiting stem, the green color of the stalk starts to turn into purplish-red, which is due to the senescent signaling of dying plant (Figure 12). After the dispersion of mericarps, the fruiting stem dries and remains in its place for at least a year. Perhaps the remnant of the dead stalk was the source of Theophrastus’ statement about the longevity of the stem of the silphion plant: “The stalk lasts only a year, like that of Ferula”. Theophrastus also suggested that the leaf of silphion is “of a golden color” [9]. Although the leaves of *F. drudeana* are green, as is the case for the other extant *Ferula* species, in late summer before they become completely dry as the leaves start losing their chlorophyll and turn into a golden color as described by Theophrastus (Figure 12).

### 3.6. Reassessment of the Silphion Extinction Event in the Cyrenaic Region

Herodotus described the limits of the “country of silphion” as starting from the island of Platea to the entrance of the Gulf of Syrtis [170], which extends approximately 350 km, yet based on the various historical records some authors expand the silphion distribution region to ca. 700–800 km by adding the area between Tobruk to El-Alamein near the Libya–Egypt border [7]. Although the stalks of *F. drudeana* always appear after the heavy spring downpour, as mentioned by Pliny the Elder’s mythical “black rain”: “We find it stated by the most trustworthy among the Greek writers (presumably Theophrastus), that this plant made its appearance near the gardens of the Hesperides and the Greater Syrtis, immediately after the earth had been soaked on a sudden by a shower as black as pitch. This took place seven years before the foundation of the city of Cyrenae, and in the year of Rome 143” [13]. Scientifically, it is impossible to claim that a plant species, that is distributed as widely as is mentioned in the historical records, appears on a single night. In contrast, the silphion plant must have been developed into its ancient glory over the hundreds of thousands of years. Regardless of its biological development history, how could a plant species that grew on such a widespread area became extinct? Pliny the Elder described a steep decline of the population of silphion plant in the Cyrenaica region and sites that “For these many years past, however, it has not been found in Cyrenaica, as the farmers of revenue who holds the lands there on lease, have a notion that it is more profitable to depasture flocks of sheep upon them. Within the memory of the present generation, a single stalk is all that has ever been found there, and that was sent as a curiosity to the Emperor Nero” [13]. In contrast to the farmers of revenue of the silphion fields, earlier owners minded silphion plants meticulously in a sustainable manner. Even Theophrastus describes the application of certain regulations during the harvest of silphion juice: “They have regulations, like those in use in mines, for cutting the root, in accordance with which they fix carefully the proper amount to be cut, having regard to previous cuttings and the supply of plant. For it is not allowed to cut it wrong nor to cut more than the appointed amount; for, if the juice is kept and not used, it goes bad and decays” [9].

**Figure 13:**
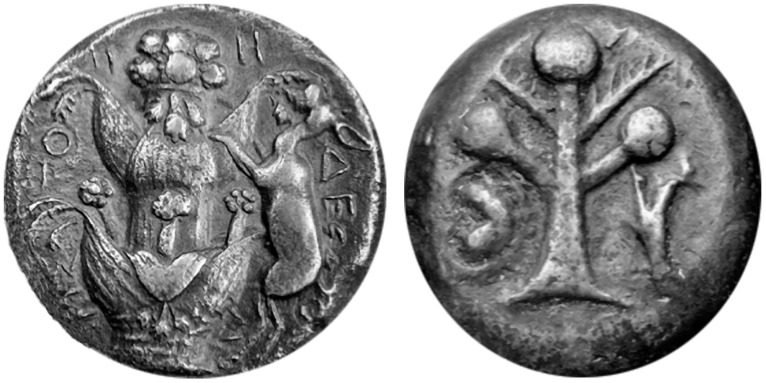
Cyrenaic coin figure depicting herbivore animals feeding on the fruits of silphion plant.

**Figure 14:**
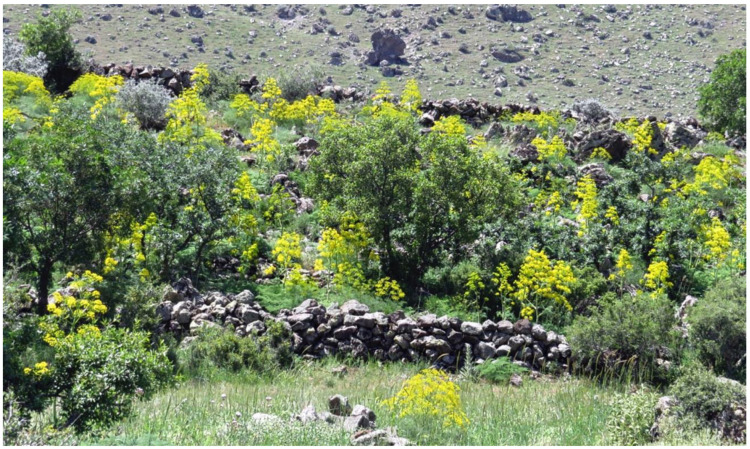
General view of the *Ferula drudeana* population in the stone-walled garden.

As was depicted by the numismatic figures (Figure 13), herbivore animals love to feed on the fruits of the silphion plant. In fact, due to consumption by livestock residing in the nearby shelter, we were not able to find a single stalk with fruits in the small population of *F. drudeana* growing in the adjacent lot of a stone-walled orchard. If the garden where *F. drudeana* is growing was not protected by the piled stone-wall (Figure 14), the *F. drudeana* population growing there might have been destroyed by now, as happened in the neighboring lot. Interestingly, Arrian (96–180 AD) mentions that in Cyrene they fence off the silphion growth sites so that the animals cannot get to them [16].

The impact of these negative ecological and man-made factors, in addition to the over harvesting of the silphion plant, driven by short-term profit, definitely contributed to the extinction of this species. However, if the silphion plant was an easily growing annual or perennial species, as mentioned by Theophrastus in the “Enquiry into Plants”: “The root and the stalk grow in the same year; nor is this a singular feature, unless they mean that it grows immediately after the dispersal of the seed since the same thing occurs with other plants also” [9] it should not have become extinct in its original location. The monocarpic nature and extremely slow growth of the *F. drudeana* likely identifies the main reason behind the extinction of this species in the Cyrenaic region of Libya.

## 4. Conclusions

The association of existing populations of *Ferula drudeana* with the locations of former Greek villages in Anatolia and morphologic comparability between the silphion plant, *F. drudeana*, and descriptions of the ancient scientists, implies a relation between the silphion plant and *F. drudeana.* Furthermore, qualitative and quantitative richness of its secondary metabolites, plausible similarities between their pharmacologic activities and medicinal usages of silphion described by the ancient authors, as well as limited archaeobotanical evidence strongly suggests that *F. drudeana*, presumably, is the silphion plant. However, until the head-to-head comparison of an archaeologic silphion sample with the extracts or resin of *F. drudeana* is conducted, we cannot confirm the identity of *F. drudeana* as being that of the silphion plant. We will continue to investigate the secondary metabolites of *F. drudeana* and try to identify suitable agricultural techniques for the effective propagation, and conservation of this invaluable species.

Thanks to the ancient smugglers who presumably have brought the seeds of the silphion plant to Anatolia [5], this precious species may have survived the extinction that its relatives had suffered in the Cyrenaic region of Libya ca. 2000 years ago. However, currently there is a greater danger lurking around *Ferula drudeana* growing in Anatolia perpetrated by the onslaught of publications appearing in various journals that the *Ferula* species growing in Anatolia has aphrodisiac qualities [63] and can help people that have erectile dysfunction problems [171,172,173]. If short-term commercial exploitation is unchecked, the very large-scale destruction of their roots certainly will drive many endemic *Ferula* species in Turkey to extinction, as has happened with the large population of *F. elaeochytris* Korovin growing on Mt. Cassius (i.e., Kel Dağı). Ironically, the same circumstances that caused the over-exploitation and extinction of the silphion plant in the Cyrenaic region 2000 years ago, also haunts its close relatives in Anatolia. Unfortunately, if subjected to this destructive scheme, the remainder of surviving silphion plants may become completely extinct.

Because of the extremely slow growth and monocarpic nature of *Ferula drudeana*, a collaborative effort that requires participation of national/international conservatory organizations, academia and multinational biotechnology companies is necessary to save this species from an impending total extinction.

## Figures and Tables

**Table 1:** Biological Activities of the Known Secondary Metabolites of *Ferula drudeana*.

Secondary Metabolite	Biological Activities
Spathulenol (**1**)	Immunomodulator [32], anti-nociceptive [64], antimicrobial [65], alleviates cardiac fibrosis [66], antioxidant [33], antiproliferative, antimycobacterial [33], anti-inflammatory [33,67], antitumor [67].
Preisocalamendiol (**3**)	Positive GABA_A_ receptor modulator [58].
Shyobunone (**4**)	Positive GABA_A_ receptor modulator [58], insect repellant and insecticide [68].
Isoshyobunone (**5**)	Positive GABA_A_ receptor modulator [58], insect repellant and insecticide [68].
Acorusnol (**8**)	Anti-inflammatory [41], germination inhibitor [40].
Teucladiol (**11**)	Cytotoxic against MCF-7 (estrogen-responsive mammalian adenocarcinoma) [43,69], MDA-MB-435 (estrogen non-responsive mammalian cancer) [43], HCT116 (colon cancer) [69] cell lines.
Chrysothol (**12**)	Cytotoxic against MCF-7 (estrogen-responsive mammalian adenocarcinoma), MDA-MB-435 (estrogen non-responsive mammalian cancer) cell lines [43].
Umbelliferone (**14**)	Anti-inflammatory [70,71], alleviates liver fibrosis [71], bone loss prevention [72], partial restoration of erectile dysfunction [73], antioxidant [74], urease inhibitor [75], anti-bacterial, anti-fungal, antidiabetic, neuroprotective, anti-cancer, molluscicidal [76].
Scopoletin (**15**)	Inhibitor of VEGF-induced angiogenesis [77], hepatoprotective [78], prevention of FGF-2-induced angiogenesis [79], antitermite [80], antimicrobial against multi-drug resistant *Pseudomonas aeruginosa* [81], acaricidal [82], antidopaminergic and antiadrenergic [83], inhibitor of acetylcholinesterase [84], inhibitor of human tumor vascularization [85], antipsychotic [86], ameliorates steatosis and inflammation in diabetic mice [87], antibacterial against multi-drug resistant clinical isolate pathogen strains [88].
Umbelliprenin (**16**)	Antigenotoxic [89], antioxidant, anti-inflammatory, lipoxygenase inhibitor [90], matrix metalloproteinase inhibitor [91], antitumor [92], cytotoxic activity against CH1 (ovarian), A549 (lung), SK-MEL-28 (melanoma) [93], M4Beu (metastatic pigmented malignant melanoma), QU-DB (large cell lung) [94], and UO31 (renal) [48] cancer cell lines, modulator of melanogenesis [95], antihypertension [96], cancer chemoprevention [97], antiangiogenic [98], antimetastatic and immunostimulatory [99].
Conferone (**17**)	Urease inhibitor [75], cytotoxic activity against CH1 (ovarian), A549 (lung) and SK-MEL-28 (melanoma) cancer cell lines [93], cancer chemoprevention [97], antiangiogenic [98].
Feselol (**18**)	Cancer chemoprevention [97], potential aphrodisiac [63].
Conferol (**19**)	Urease inhibitor [75], modulators of multi-drug resistance in clinical isolates of *Escherichia coli* and *Staphylococcus aureus* [100], antileishmanial [101], antiviral against Influenza A (H_1_N_1_) virus [102], cytotoxic against HepG2 (hepatocellular carcinoma), Hep3B (hepatocellular carcinoma) and MCF-7 (estrogen-responsive mammalian adenocarcinoma) cancer cell lines [102].
Badrakemone (**20**)	Cytotoxic against UO31 (renal) cancer cell line [48], cancer chemoprevention [97], weak matrix metalloproteinase inhibitor [91].
Colladonin (**21**)	Cytotoxic against COLO205 (colon), KM12 (colon), A498 (kidney carcinoma), UO31 (renal), TC32 (Ewing’s sarcoma) [48], HCT116 (human colorectal), HT-29 (human colorectal) [103] cancer cell lines, endocannabinoid system modulator [104].
Badrakemin (**22**)	Cytotoxic against KM12 (colon), A498 (kidney carcinoma), UO31 (renal) cancer cell lines [48], inhibitor of butyrylcholinesterase [105].
Samarcandin (**23**)	Potential aphrodisiac [63], antifungal [54], cytotoxic against AGS (human gastric carcinoma), WEHI-164 (fibrosarcoma) cancer cell lines [106], active in NCI yeast anticancer drug screen assays [107], potential antiviral activity against Ebola virus [108,109].
Samarcandin Acetate (**26**)	Potential aphrodisiac [63].
Myristicin (**27**)	Cancer chemopreventive agent [51], anti-inflammatory [52], antimicrobial against *B. subtilis*, *E. coli*, *S. aureus* [110], hepatoprotective [111], cytoprotective against hypoxia-induced apoptosis and endoplasmic reticulum stress [112], down-regulates expression of pro-inflammatory cytokines [113], cytotoxic against SK-N-SH (human neuroblastoma) cancer cell line [114], acetylcholinesterase inhibitor [115], antiproliferative [116], insecticidal against *Culex pipiens* and *Aedes aegypti* [117].
Laserine (**28**)	Weak cytotoxicity against HL-60 (acute promyelocytic leukemia) cancer cell line [118], inhibitor of skin photo-aging [119].
Cynaroside (**29**) (Luteolin-7-β-d-glucoside)	Choleretic and anticholestatic [120], antioxidant and anticholinesterase [121], antibacterial against multi-drug resistant clinical isolate strains [88], anti-inflammatory [122], inhibitor of monoamine oxidase B [123], inhibitor of low-density lipoprotein (LDL) oxidation [124], antimicrobial [125], hepatoprotective [126].
Cynarin (**30**)	Antimicrobial [125], hepatoprotective [126], antihypertensive, vasodilator [127], choleretic [128].
Chlorogenic Acid (**31**)	Antimicrobial [125], hepatoprotective [126], antihypertensive, vasodilator [127], antitumor [129], anti-inflammatory [130], improves late diabetes [131], protects against cholestatic liver injury [132], neuroprotective [133], antiviral activity against influenza A (H1N1/H3N2) virus [134], anti-diabetic and anti-lipidemic [135], inhibits hepatocellular carcinoma [136], anxiolytic and antioxidant [137], antihyperalgesic [138], cardioprotective [139], neuroprotective and cognitive improvement [140], improves hepatic steatosis and insulin resistance [141], alleviates obesity and modulates gut microbiota [142], ameliorates ulcerative colitis [143], inhibits glioblastoma growth [144], induces 4T1 breast cancer tumor’s apoptosis [145], strong matrix metalloproteinase-9 inhibitor [146].

## Data Availability

The data presented in this study are available on request from the corresponding author.
